# Case report: Dual-targeted BCMA and CS1 CAR-T-cell immunotherapy in recurrent and refractory extramedullary multiple myeloma

**DOI:** 10.3389/fimmu.2024.1422478

**Published:** 2024-07-30

**Authors:** Xiangjun Shi, Yue Wu, Xingchen Yao, Boran Du, Xinru Du

**Affiliations:** ^1^ Department of Rheumatology and Immunology, Beijing Tiantan Hospital, Capital Medical University, Beijing, China; ^2^ Department of Orthopedics, Beijing Chaoyang Hospital, Capital Medical University, Beijing, China; ^3^ Department of Pharmacy, Capital Medical University, Beijing Obstetrics and Gynecology Hospital, Beijing, China

**Keywords:** BCMA, CS1, CAR-T, multiple myeloma, immunotherapy

## Abstract

**Background:**

The development of CAR-T-cell immunotherapy has notably elevated the efficacy of treating multiple myeloma. Currently, a variety of targets, including BCMA, CS1, CD38, FcRH5, and GPRC5D, are being investigated. Despite these significant advancements, challenges such as antigen escape, limited persistence of CAR-T cells, and the intricate nature of the tumor microenvironment persist, leading to relapses following treatment.

**Case presentation:**

We report the case of a patient with recurrent and refractory multiple myeloma (RRMM) who developed a substantial extramedullary plasmacytoma in the muscles of the lower limb following multiple rounds of radiotherapy and chemotherapy. The patient underwent CAR-T-cell immunotherapy targeting BCMA and CS1; however, the tumor progressed despite treatment. Surgical resection of the extramedullary plasmacytoma was subsequently performed. Upon comparison of the tumor tissue with the adjacent tissue, increased expression of MYBL2 was noted in the tumor tissue, potentially contributing to the lack of improvement in extramedullary relapse after dual-targeted CAR-T cell therapy.

**Conclusions:**

In patients with recurrent and refractory multiple myeloma who underwent multiple cycles of chemotherapy and radiotherapy, dual-targeted CAR-T cell therapy aimed at BCMA and CS1 failed to effectively manage extramedullary relapse. Elevated expression of MYBL2 in multiple myeloma correlates with a poorer prognosis.

## Introduction

CAR-T cell immunotherapy for multiple myeloma has improved treatment efficacy and prolonged survival to some extent (1). However, relapse after CAR-T cell therapy often indicates a poor prognosis, especially in patients with refractory myeloma or those with extramedullary plasmacytoma. B-cell maturation antigen (BCMA) has emerged as a novel target for treating multiple myeloma (MM) due to its highly selective expression in malignant plasma cells (PCs) (2). Various BCMA-targeted treatment approaches, including antibody−drug conjugates (ADCs), chimeric antigen receptor (CAR) T cells, and bispecific T-cell engagers (BiTEs), have shown significant clinical efficacy in relapsed and refractory MM patients (3). CS1 is also highly expressed in myeloma and has shown effective therapeutic outcomes in clinical treatment with humanized elotuzumab and Empliciti (4, 5). However, adverse reactions following CAR-T cell therapy have been increasing annually, leading the FDA to mandate warnings about the induction of secondary cancers, including T-cell malignancies posttargeted CD19 and BCMA CAR-T cell therapies, on the market (6). Nonetheless, data on failed CAR-T cell therapy remain insufficient (7). This article focuses on the case of a patient with refractory/relapsed multiple myeloma accompanied by extramedullary plasmacytoma, where targeted CAR-T cell therapy directed at the BCMA and CS1 failed to provide relief, leading to subsequent surgical pathology results and exploration of related prognostic factors (8).

## Case presentation

An adult patient presented with persistent right nasal congestion in 2015, with occasional rhinorrhea and sneezing, accompanied by headaches, a normal sense of smell, no asthma, and normal vision. In October 2016, bilateral purulent rhinorrhea with blood-tinged discharge appeared without obvious cause and was more severe on the right side, prompting a medical consultation. The patient had a history of Sjögren’s syndrome for more than 10 years and is currently experiencing symptoms of dry mouth and dry eyes. In 2008, patient underwent surgical treatment for pneumonia-associated plasmacytoma. Imaging examination: A sinus CT scan revealed soft tissue shadows in the right nasal vestibule and nasal cavity, suggesting a nasal mass. Pathological examination of the mass biopsy indicated extramedullary plasmacytoma (differentiated type); immunohistochemistry revealed CD79a+, CD38+, CD138-, κ+, λ-, CD20-, CD3-, LCA-, CK+, Vimentin+, EMA+, P63-, CD68-, S-100-, P53-, and Ki67 at 50%, and CD34+, ChgA-, Syn-, and NSE-. The diagnosis was intranasal extramedullary plasmacytoma. Following diagnosis, the patient received 25 weeks of nasal radiotherapy (2 Gy/session/week, total dose 50 Gy). In January 2017, the patient experienced sternal pain without obvious cause or fatigue. A PET-CT scan performed on June 5, 2017, revealed multiple bone lesions with bone destruction and thickening of the surrounding cartilaginous tissue in the sternum, third thoracic vertebra, left second anterior rib, and right eighth posterior rib, with abnormal radioactive concentrations. Sternum puncture confirmed plasmacytoma, with immunohistochemistry showing plasma cell origin: CD20-, CD3-, CD38+, LCA-, Ki67 + 20%, MUM1+, CD56-, CyclinD1-, CD117-, κ+, λ-. Immunoglobulin levels were as follows: IgG, 43 g/L; IgA, 10.31 g/L; κ, 11.8 g/L; and λ, 4.32 g/L, negative for M protein. Bone marrow morphology: mature plasma cells at 1%. Flow cytometry of the bone marrow did not reveal abnormal plasma cell phenotypes, and bone marrow biopsy revealed no abnormal plasma cell proliferation. Diagnosis: Multiple myeloma IgA κ+, staging: RISS. Treatment included chemotherapy with a TD regimen beginning on June 30, 2017, consisting of thalidomide (200 mg for 1–14 days) and bortezomib (30 mg for 1–14 days). On August 18, 2017, spinal MRI revealed abnormal signals in the sixth thoracic vertebra, sacral vertebrae 3–5, with changes suggestive of posttreatment, and absorptive destruction in the spinous process of the third thoracic vertebra and the sternal manubrium, indicating malignant changes. Whole-body DWI revealed a high signal in the sternal manubrium, third thoracic vertebra, liver, and rectal area. Completion of two cycles of the TD regimen by September 4, 2017. On September 6, 2017, his urine immunoglobulin L light chain levels were as follows: κ<1.85 mg/dL, λ<5 mg/dL; blood immunoglobulin IgG, 1280 mg/dL; IgA, 322 mg/dL; IgM, 38.9 mg/dL; IgE<17.7 IU/ml; and κ, 1190 mg/dL, λ, 583 mg/dL. Chest CT revealed the formation of plasmacytomas in the third and fourth anterior ribs, sternum, and eighth posterior rib. The PAD regimen (bortezomib, doxorubicin, dexamethasone) was administered on October 12, 2017. Fixed electrophoresis on November 6, 2017, revealed IgA and κ monoclonal bands, and his urine Bence-Jones protein test was negative. The PACD regimen was administered on November 7, 2017. Fixed electrophoresis on December 4, 2017, revealed IgA and κ monoclonal bands, and his urine Bence-Jones protein test was negative. The second cycle of the PACD regimen was administered on December 8, 2017. The PCDD regimen was started on January 3, 2018 (2.2 mg melphalan on Days 1, 4, 8, and 11; 300 mg cyclophosphamide on Days 1, 4, 8, and 11; 20 mg dexamethasone on Days 1, 2, 4, 5, 8, 9, 11, and 12; and 40 mg doxorubicin on Day 4). Bone radiotherapy was administered from January 29 to March 6, 2018.

In April 2018, a round, tough, immobile mass with a diameter of 3 cm was found in both thighs, without tenderness, distal radiating pain, or numbness. Immunohistochemistry showed positive reactions for CS1 and BCMA. Tissue FISH analysis suggested possible P53 gene deletion, a 1q21 gene amplification, an IgH gene deletion (or -14), an FGFR gene deletion (or -4), and a CCND1 gene deletion (or -11). Mutation testing of lymphoid tissue tumors revealed a splice site mutation in the TP53 gene, c.375 + 1G>A, which suggests a poor prognosis for the patient. A missense mutation in NRAS gene exon 3, c.182A>T, p.Q61L, was associated with a reduced treatment response to bortezomib and a shorter disease progression period. Missense mutations related to high-risk disease progression were detected in the KMT2D gene, c.1672C>G, p.G558A; the BCL2 gene, c.23G>C, p.G8A; and the IGLL5 gene, c.82T>A, p.C28S. Insertion and complex mutations were found in exons 7 and 9 of the TET2 gene. A missense mutation was discovered in the FAT1 gene, c.10989C>A, p.F3663L.

On April 16, 2018, immunohistochemistry of subcutaneous tissues showed positive reactions for CS1 and BCMA. Lymphocyte collection was performed for CAR-T cell preparation, and the patient was concurrently treated with idelalisib and cyclophosphamide, along with symptomatic supportive therapies, including organ protection, hydration, and antiemetics. Starting on May 4, 2018, the patient received fludarabine and cyclophosphamide pretreatment, followed by BCMA CAR-T cell infusion on May 12, 2018, with a total cell count of 5.3×10^8 and cell viability of 91.3% at an effective cell count of 2.2×10^6/kg based on body weight. CS1 CAR-T cells were infused on May 13, 2018, with a total cell count of 3.3×10^8, a cell viability of 94.4%, and an effective cell count of 1.0×10^6/kg based on body weight.

**Figure 1:**
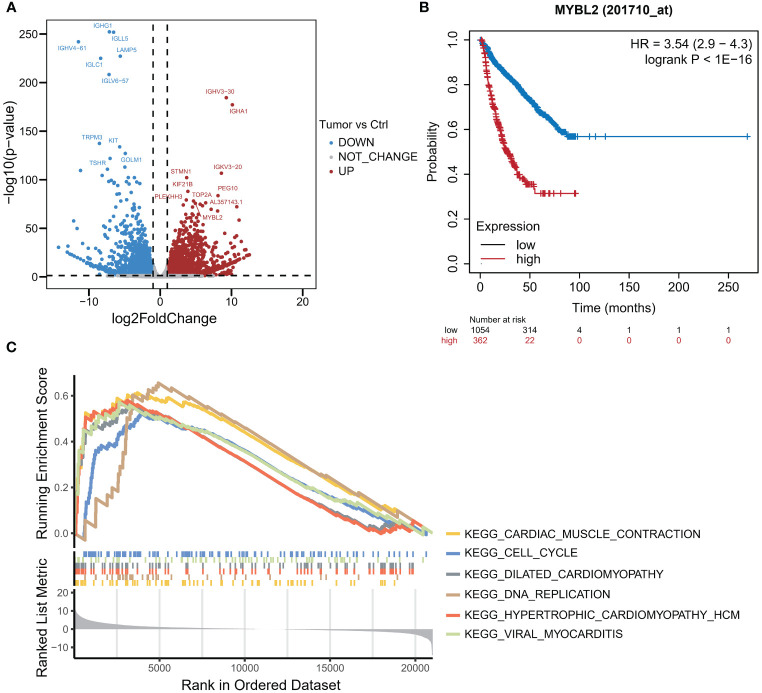
RNA-seq analysis of MM tumor and paracarcinoma tissues. **(A)** Volcano plot of differential mRNA expression between tumor and paracarcinoma tissues according to RNA-seq. **(B)** K−M curve of MM samples from the TCGA dataset. **(C)** The results of gene set enrichment analysis (GSEA).

**Figure 2:**
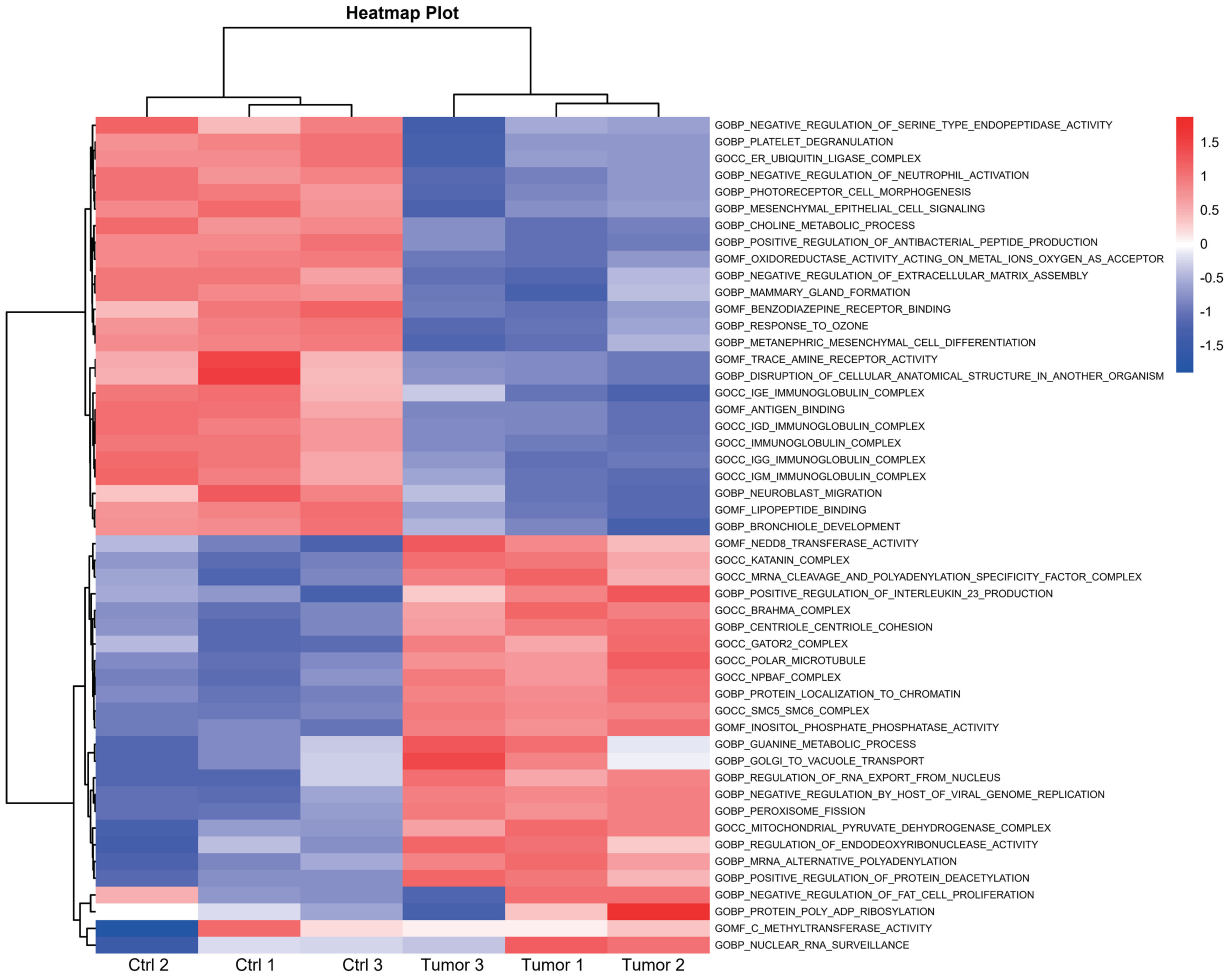
Heatmap illustrating the pathways associated with DEGs between the high- and low-risk subgroups according to GSVA.

**Figure 3:**
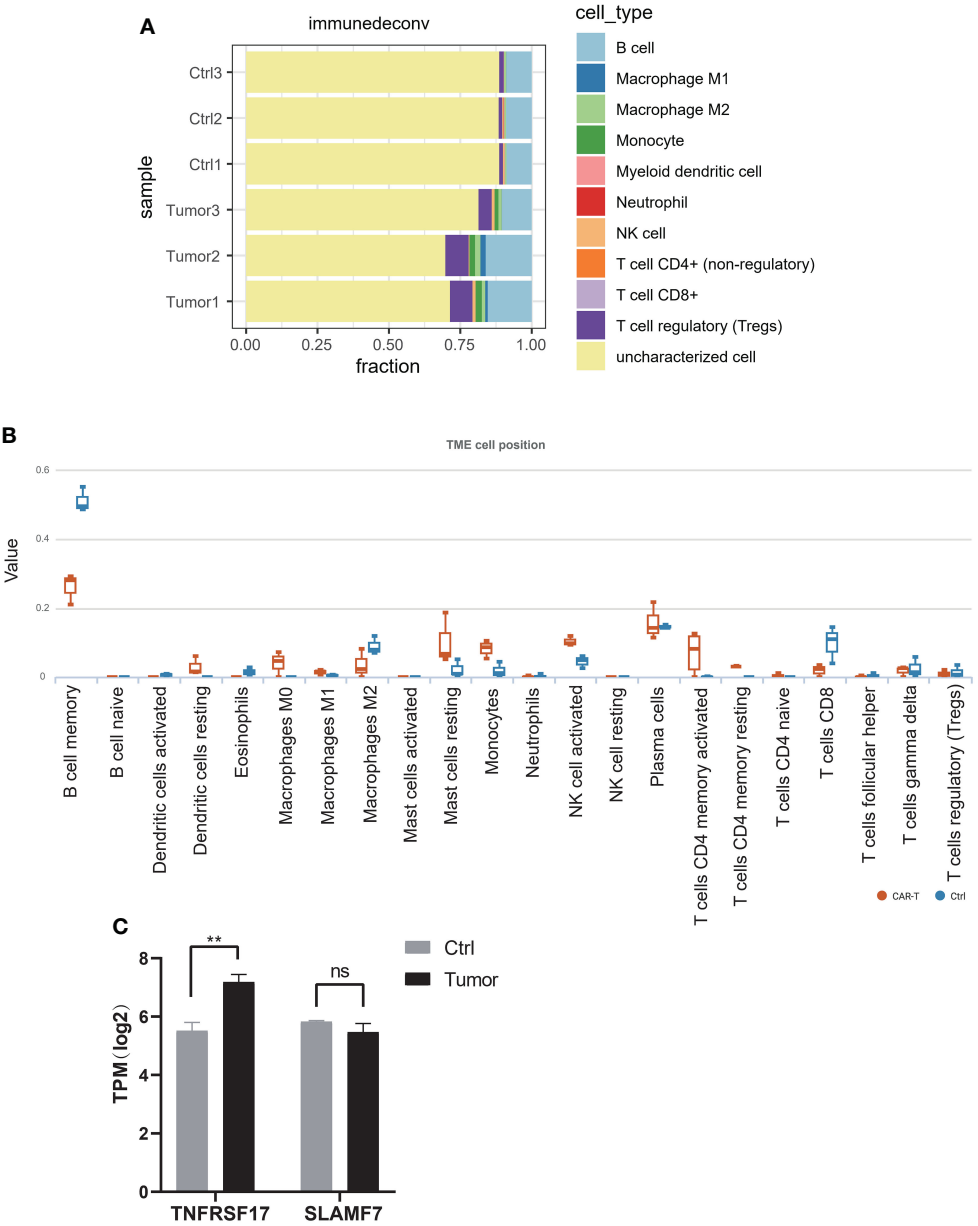
**(A)** Immune cell infiltration was analyzed using the MCP-counter algorithm in the R package “immunedeconv”. **(B)** The CIBERSORT algorithm was used for immune infiltration analysis to compute the proportions of 22 immune cell types, and tables and box plots were generated. **(C)** The expression of TNFRSF17 and SLAMF7 according to RNA-seq. *p<0.05, **p<0.01, ***p<0.01. ns, not significant.

In July 2018, the mass gradually increased to approximately 20 cm × 30 cm × 30 cm in size, with tenderness and radiating pain in the right thigh mass. The nature of the left thigh mass remained similar to before. The patient was diagnosed with multiple myeloma and extramedullary plasmacytoma. On July 9, 2018, the tumor in the right thigh was surgically removed. Tumor and peritumoral tissues were collected during surgery for RNA sequencing (RNA-seq) analysis. Genes with log2FC>1.5 and p<0.05 were obtained, resulting in 2323 upregulated genes and 1930 downregulated genes (**Figure 1A**). The differentially upregulated genes included IGHV3–30, IGHA1, IGKV3, STMN1, PEG10, KIF21B, PLEKHH3, TOP2A, AL357143.1, and MYBL2. The differentially downregulated genes included IGHG1, IGLL5, LAMP5, IGHV4–61, IGLC1, IGLV6–57, TRPM3, KIT, TSHR, and GOLM1. Prognostic analysis of MYBL2 in myeloma using Kaplan−Meier database plotting tools revealed a correlation with reduced survival in myeloma patients (**Figure 1B**). GSEA indicated enrichment of pathways related to cardiac muscle contraction, the cell cycle, dilated cardiomyopathy, DNA replication, hypertrophic cardiomyopathy, and viral myocarditis in tumor tissues compared to adjacent tissues (**Figure 1C**). GSVA revealed significant upregulation of the IL-23 generation signaling pathway, which has been associated with reduced infiltration of CD8+ T cells in the tumor microenvironment and inhibitory effects on T-cell responses (**Figure 2**). Additionally, a significant downregulation of the immunoglobulin IgE, IgG, IgD, and IgM protein complex signaling pathways was observed, suggesting a decreased ability of multiple myeloma cells to secrete immunoglobulins within the tumor, potentially indicating increased proliferative capacity. Immune infiltration analysis using an immune system revealed a significant increase in regulatory T cells and both M1 and M2 macrophages in the tumor immune infiltration group compared to those in the control group. Furthermore, CIBERSORT algorithm analysis demonstrated a reduction in memory B cells in MM patients, and GSVA results indicated decreased immunoglobulin levels in MM patients. Additionally, an increase in resident dendritic cells, macrophages, mast cells, mononuclear cells, and activated memory CD4^+^ T cells and a decrease in cytotoxic CD8^+^ T cells were observed in myeloma patients (**Figures 3A, B**). BCMA (TNFRSF17) was found to be highly expressed in tumor tissues, while CS1 (SLAMF7) showed no significant difference in expression compared to adjacent tissues (**Figure 3C**).

## Discussion

CAR-T cell therapy has emerged as a highly promising approach for treating cancer, with CAR-T cell exhaustion widely recognized as a major limiting factor for its efficacy. Addressing CAR-T cell exhaustion to achieve sustained immunotherapeutic effects has garnered considerable attention (9). However, intrinsic tumor factors influencing CAR-T immunotherapy outcomes have also piqued scholarly interest. Autologous CD19 CAR-T cells have demonstrated significant efficacy in treating relapsed and refractory diffuse large B-cell lymphoma (DLBCL), with three products receiving approval from the US FDA. Response rates range from 50% to 80%, yet over half of patients experience disease relapse and progression. Elucidating factors contributing to disease relapse and progression can guide combination therapies or the development of alternative consolidation strategies. Current research predominantly focuses on patient serum biomarkers, T cell exhaustion, and factors associated with adverse prognosis. Intrinsic tumor factors also play a crucial role in adverse prognosis events (10, 11). Cherng et al. observed long-term remission in relapsed/refractory DLBCL patients following CD19 CAR-T cell therapy, although more than half experienced treatment failure. They developed a minimally invasive detection method based on whole genome sequencing results to quickly identify high-risk patients (12). Jain et al. identified tumor-intrinsic genomic alterations as key factors influencing resistance to CAR-T 19 therapy in DLBCL (13).

Two CAR-T cell therapy products targeting BCMA, idecabtagene vicleucel (ide-cel) and ciltacabtagene autoleucel (cilta-cel), have received approval from the US FDA for treating relapsed/refractory multiple myeloma patients. These patients have typically undergone four or more prior lines of therapy, including immunomodulatory drugs, proteasome inhibitors, and anti-CD38 monoclonal antibodies. While most patients achieve responses, some experience relapse, and the mechanisms of resistance remain incompletely understood (14). SLAMF7, also known as CS1, is a gene of the SLAM family highly expressed in plasma cells but not in non-hematopoietic tissues. Humanized monoclonal antibodies targeting SLAMF7 in combination with lenalidomide have achieved higher response rates in MM (15). Studies suggest that CAR-T cells targeting both BCMA and CS1 exhibit superior anti-myeloma effects compared to CAR-T cells targeting BCMA alone (16).

A clinical trial registered on clinicalTrials.gov (NCT04662099) yielded promising results for CS1-BCMA CAR-T cell therapy. Among 16 relapsed or refractory MM patients who received treatment, 3 had no response in extramedullary MM, while 6 achieved complete remission. However, 4 patients experienced BCMA+ and CS1+ relapse or progression. This study demonstrated good clinical activity and safety of CS1-BCMA CAR-T cells in RRMM patients. Importantly, no progression of extramedullary plasmacytoma has been reported (7, 8, 17).

Patients who received both BCMA CAR-T cell therapy and CS1 CAR-T cell therapy had high preoperative expression of both targets (18). Postoperative RNA-Seq results showed sustained high expression of BCMA in tumor tissue but no significant change in CS1 expression. Extramedullary plasmacytoma continued to progress rapidly, possibly due to the suppression of the T-cell response by reducing CD8^+^ T-cell infiltration in the tumor microenvironment through binding with Th17-associated proinflammatory cytokines. Additionally, downregulation of the IgE, IgG, IgD, and IgM protein complex signaling pathways indicated a decreased ability to secrete tumor-infiltrating immunoglobulin, enhancing proliferation and potentially contributing to rapid tumor recurrence after CAR-T cell therapy (19).

In RRMM patients treated with CS1-BCMA CAR-T cell therapy, 6 out of 16 patients achieved complete remission, indicating efficacy for certain patients, despite treatment failure or relapse. Further research is needed to explore the reasons for treatment failure or relapse and improve treatment strategies. While both BCMA and CS1 are highly expressed in multiple myeloma tissues, postoperatively, BCMA antigen remained highly expressed, whereas CS1 expression decreased. This finding suggested that CS1 expression may be regulated by other factors or may be minimally affected by treatment. Future studies can explore the expression mechanisms of BCMA and CS1 and their roles in CAR-T cell therapy (17).

MYBL2 belongs to the MYB gene family, which also includes MYB and MYBL1. Research indicates that MYB proteins play a crucial role in supporting cancer cell survival, thereby constituting a major obstacle in cancer therapy. MYB proteins are involved in various oncogenic processes, including cell proliferation, differentiation, survival, invasion, and extracellular matrix remodeling (20, 21). These proteins can sustain cancer cell growth under adverse conditions and confer resistance to treatment, making MYB a potential therapeutic target in cancer treatment. MYBL2 plays a significant role in regulating cell proliferation, differentiation, and DNA repair. Studies by Bayley et al. have demonstrated that elevated MYBL2 expression correlates with metastasis, poorer disease-free survival, and shorter overall survival in breast cancer, highlighting how increased MYBL2 expression contributes to the disease’s enhanced invasiveness (22, 23).

This study revealed the impact of IL-23 on the multiple myeloma microenvironment, which may affect CD8^+^ T-cell infiltration and the antitumor effect of CAR-T cells (24). Understanding the role of IL-23 in this context can provide new insights for enhancing the efficacy of CAR-T cell therapy (25).

Furthermore, the downregulation of the immunoglobulin signaling pathway and the decreased ability of multiple myeloma cells to secrete immunoglobulins within the tumor, along with enhanced proliferative capability, suggest a potential link between immunoglobulin signaling and tumor recurrence. Further research is necessary to investigate these pathways in MM and their relationship with the efficacy of CAR-T cell therapy (26).

In conclusion, this study provides preliminary insights into the clinical effects and mechanisms of CS1-BCMA CAR-T cell therapy in relapsed/refractory multiple myeloma patients. Further validation and refinement of these findings through additional research are crucial for guiding future treatment strategies.

## Data availability statement

The datasets presented in this study can be found in online repository. The name of the repository and accession number can be found here: PRJNA1137965 (BioProject). http://www.ncbi.nlm.nih.gov/bioproject/1137965.

## Ethics statement

The studies involving humans were approved by Beijing Chaoyang Hospital Ethics Committee (2018ke111). The studies were conducted in accordance with the local legislation and institutional requirements. The participants provided their written informed consent to participate in this study. Written informed consent was obtained from the individual(s) for the publication of any potentially identifiable images or data included in this article.

## Author contributions

XS: Funding acquisition, Writing – original draft. YW: Writing – original draft. XY: Data curation, Writing – original draft. BD: Data curation, Writing – original draft. XD: Investigation, Writing – review & editing.
